# The Automation of Breast Ultrasonography and the Medical Time Dedicated to the Method

**DOI:** 10.1055/s-0043-1772176

**Published:** 2023-08-18

**Authors:** Katyane Larissa Alves, Ruffo Freitas-Junior, Régis Resende Paulinelli, Marcus Nascimento Borges

**Affiliations:** 1 Universidade Federal de Goiás, Goiânia, GO, Brazil

**Keywords:** mammary ultrasonography, breast ultrasonography, diagnostic imaging, breast neoplasms, three-dimensional imaging, ultrassonografia mamária, diagnóstico por imagem, neoplasias da mama, imageamento tridimensional

## Abstract

In this integrative review, we aimed to describe the records of time devoted by physicians to breast ultrasound in a review of articles in the literature, in order to observe whether the automation of the method enabled a reduction in these values. We selected articles from the Latin American and Caribbean Literature in Health Sciences (LILACS) and MEDLINE databases, through Virtual Health Library (BVS), SciELO (Scientific Electronic Library Online), PubMed, and Scopus. We obtained 561 articles, and, after excluding duplicates and screening procedures, 9 were selected, whose main information related to the guiding question of the research was synthesized and analyzed. It was concluded that the automation of breast ultrasound represents a possible strategy for optimization of the medical time dedicated to the method, but this needs to be better evaluated in comparative studies between both methods (traditional and automated), with methodology directed to the specific investigation of this potentiality.

## Resumo

Na presente revisão integrativa, objetivamos descrever os registros de tempo dedicado pelos médicos à ultrassonografia mamária em revisão de artigos da literatura, visando observar se a automação do método possibilitou redução destes valores. Selecionamos artigos nas bases de dados Literatura Latino-Americana e do Caribe em Ciências da Saúde (LILACS) e MEDLINE, através da Biblioteca Virtual em Saúde (BVS), *Scientific Electronic Library Online* (SciELO), PubMed e Scopus. Obtivemos 561 artigos e, após a exclusão de artigos duplicados e procedimentos de triagem, foram selecionados 9 artigos, cujas informações principais relativas à pergunta norteadora da pesquisa foram sintetizadas e analisadas. Foi concluído que a automação da ultrassonografia mamária representa uma possível estratégia de otimização do tempo médico dedicado ao método; porém, essa conclusão necessita ser melhor avaliada em estudos comparativos entre ambos os métodos (tradicional e automatizado), com metodologia direcionada à investigação específica desta potencialidade.

## Introduction

The optimization of the medical workflow, while maintaining the accuracy of diagnostic methods, has been observed among the objectives of studies related to breast ultrasound. In its traditional form, breast ultrasound requires a medical time that is usually considered long. 1 2 3

In this context, automated breast ultrasound was developed, initially aiming at reducing the medical time for evaluating the ultrasound images, transferring the acquisition time of the same to a radiology technician, with specific training, allowing the use of the method on a large scale, for breast cancer screening. 1 4 5

The automated breast ultrasound device has a larger transducer than the conventional one, coupled to a mechanical arm, performing an automatic and standardized scan of the entire breast. The images obtained are transferred to a workstation where they are available for medical interpretation. 6 7 Three images are obtained (anteroposterior, lateral and medial of each breast), forming three planes or views for interpretation: coronal, sagittal, and transverse. 8 9

Factors such as the learning curve of the automated method, the physicians' experience with each of the methods, the number of findings, the size of the breasts (since a greater amount of breast tissue may require acquisition of additional views in the automated method and represents greater tissue volume to be evaluated also in the conventional method), interfere in this measure of time in an already established way. 3 8 10 11

The evaluation of the coronal view only, with the objective of reducing the time required for the physician to interpret the automated images, was analyzed by Schiaffino et al. Therefore, the multiplanar evaluation is mandatory, that is, all images must be obtained for a good diagnostic performance. 12

The use of computer algorithm systems to help detect changes in images obtained by automated ultrasound (computer-aided detection [CAD] system) is another strategy that has also been analyzed in some studies, with a reduction in medical interpretation time using these algorithms. 7 13

Thus, we aimed to describe the records of time dedicated by physicians to breast ultrasound in a review of literature articles, in order to observe whether the automation of the method made it possible to reduce these values.

## Methods

This is an integrative literature review, developed observing the following steps: elaboration of the research question, selection of literature articles, data extraction and critical analysis of the included articles, presentation and discussion of the results obtained, and establishing the conclusion of the authors. 14

To define the question to be answered with the search for articles, the patient, intervention, comparison, and outcomes (PICO) strategy was used. 15 Our research object was the medical time required for breast evaluation using the automated way of obtaining the images. The intervention was defined as the use of the automated method of ultrasound of the breasts and our comparison was established with the conventional method of performing this exam, with the expectation as an outcome to reduce this medical time with the use of the automated method. Thus, we used the following question to guide our review: How long does the physician need to evaluate the automated ultrasound images of the breasts? Would this time be shorter than the time required to perform a conventional (non-automated) ultrasound of the breasts?

The selection of articles was made in July and August of 2022 in the Latin American and Caribbean Literature in Health Sciences (LILACS) and MEDLINE databases, through the Virtual Health Library (BVS), Scientific Electronic Library Online (SciELO), PubMed, and Scopus. As descriptors, in Health Sciences (DeCS) and Medical Subject Headings (MeSH), we used *mammary ultrasonography* , *breast ultrasonography* , *diagnostic imaging* , *breast neoplasms* , and *three-dimensional imaging* .

We applied language filters, selecting articles in English and Portuguese, with full text available, and selected screening, diagnosis, prognosis, evaluation, and observational studies in the areas of medicine, imaging, gynecology, and radiology as the type of studies.

## Results

**Fig. 1:**
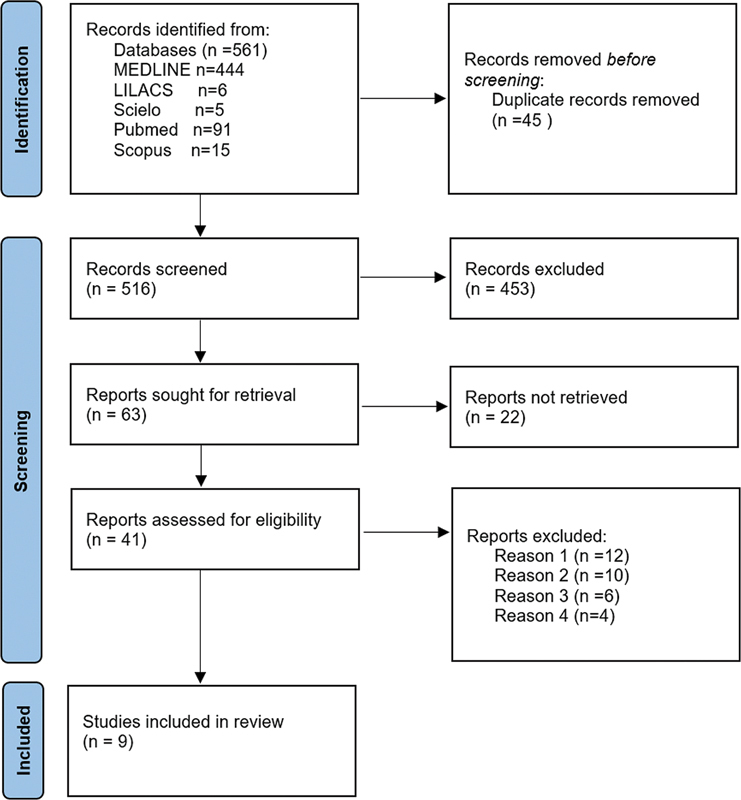
PRISMA 2020 flowchart with database search results. *From:* Page MJ, McKenzie JE, Bossuyt PM, Boutron I, Hoffmann TC, Mulrow CD, et al. The PRISMA 2020 statement: an updated guideline for reporting systematic reviews. BMJ 2021;372:n71. doi: 10.1136/bmj.n71

We obtained 561 articles from the databases, and, using the Rayyan application (Qatar Computing Research Institute, Ar-Rayyan, Qatar) 16 , 45 duplicate articles were found, leaving 516 articles for analysis. Of these, 453 were excluded and 63 were included by reading the title. Of the 63 included, 22 were excluded, and 41 were included after reading the abstract. These 41 included articles were then considered for full text reading. After reading the full text, 32 were excluded, 12 of which did not present the measurement of the medical time spent interpreting the images obtained by automated breast ultrasound (reason 1), 10 in relation to the time to perform the conventional breast ultrasound (reason 2) and 6 for both methods (reason 3), and 4 for being narrative review articles (reason 4). The remaining 9 articles provided data for the composition of Charts 1 , 2 , and 3 . 1 2 10 11 13 17 18 19 21 Figure 1 summarizes these results in the PRISMA 2020 flowchart. 22

**Chart 1:** Summary of comparative studies of medical time in both methods

Authors Characterization	Philadelpho et al.	Tutar et al.
Article title	Comparison of Automated Breast Ultrasound and Hand-Held Breast Ultrasound in the Screening of Dense Breasts	Comparison of automated versus hand–held breast US in supplemental screening in asymptomatic women with dense breasts: is there a difference regarding woman preference, lesion detection and lesion characterization?
Country/year of publication	Brazil/2021	Turkey/2020
Type of study/level of evidence	Cross-sectional study/level 4	Cross-sectional study/level 4
Sample/inclusion criteria	440/asymptomatic women with dense breasts on mammography	340/women with dense breasts and normal mammograms
Exclusion criteria	Women with breast surgery for cancer or benign causes (including implants) and/or breast radiotherapy in the last 12 months	Women at high risk and/or with suspicious clinical findings and/or with a history of breast cancer
Objectives	Comparing conventional ultrasonography with ultrasonography automated breasts in breast cancer screening	Compare ABUS and HHUS in terms of workflow, patient preference, effectiveness in detecting and characterizing lesions
Metodology	HHUS first and ABUS next (independent evaluation) HHUS: breast radiologists (n = 13) and non-specialized (n = 17) ABUS: breast radiologists only (n = 6)	ABUS first and HHUS in the sequence HHUS: breast radiologists only (n = 2) bilateral breast and underarm examination ABUS: assessment by both breast radiologists in consensus
Conclusions	Compared to HHUS, ABUS allowed adequate complementary study in the breast cancer screening	No significant differences in lesion detection, lower PPV with ABUS, more than 50% of patients prefer HHUS
Time HHUS Breast Radiologists	7 min e 45 s	12.5 min
Time HHUS non-specialist radiologists	4 min e 15 s	------------
Time ABUS breast radiologists	4 min e 25 s	14.5 min
*p* -value	*p* < 0.001 *	-----------

*Student t-test (difference between mean time of breast radiologists for HHUS and ABUS).

**Chart 2:** Summary of non-comparative studies that reported the medical time spent using the automated method

AUTHOR/ YEAR	TYPE OF STUDY/LEVEL OF EVIDENCE	SAMPLE/ INCLUSION CRITERIA	EXCLUSION CRITERIA	METHODOLOGY	AVERAGE TIME ABUS
**Skaane et al., 2015**	Retrospective study/ level 4	90 included symptomatic patients or those with clinical or mammographic alterations	Did not restrict patient participation	ABUS evaluated by 5 breast radiologists	9 min
**Wilczek et al., 2016** **(Easy Study)**	Randomized clinical trial/ Level 2	1,668 included patients aged ≥ 40 years, asymptomatic, with dense breasts	Pregnant or lactating women with a history of breast surgery and/or diagnosis and/or treatment of breast cancer in the last 12 months were excluded.	The ABUS images were analyzed by 5 breast radiologists, after evaluating the corresponding mammography	5–7 min
**Vourtsis e Kachulis 2017**	Non-randomized clinical trial/ level 3	1,886 patients symptomatic or not, with dense breasts	Did not restrict patient participation	ABUS images evaluated after respective mammograms, when available according to the case, by 2 breast radiologists	3 min
**Jiang et al., 2018**	Retrospective study/ level 4	185 patients with dense breasts	Patients submitted to previous breast interventions	18 breast radiologists interpreted the ABUS images twice (with and without the aid of computer-CAD systems)	3.5 min (without CAD) 2 min and 24 s (with CAD)

**Chart 3:** Summary of non-comparative studies that reported the medical time spent using the conventional method

AUTHOR/YEAR CHARACTERIZATION	Berg et al., 2008 (ACRIN 6666)	Chang et al., 2015	Phalak et al., 2018
Type of study/level of evidence	Randomized clinical trial/2	Retrospective study/4	Cross-sectional study/4
Sample/inclusion criteria	2,725 women at high risk for breast cancer with at least heterogeneously dense breasts in at least 1 quadrant. Patients undergoing breast cancer follow-up could be included	1,526 asymptomatic women	100 patients with a history of lobular neoplasia
Exclusion criteria	excluded women with signs or symptoms of breast cancer, with surgery, or breast intervention procedures or breast exams less than 11 months ago, pregnant women, breastfeeding women, with breast implants, with metastatic cancer	Women with a personal or family history of breast cancer and/or suspicious MMG findings	Patients with > 20% risk for breast cancer by risk models and/or with breast cancer
Methodology	USG performed by radiologists. Axillary assessment could be included and added to the total exam time	USG performed by breast radiologists. Axillary assessment routinely included in the exam and added to the total exam time	USG performed by technologists and images reviewed by radiologists. If necessary, a breast radiologist would redo the exam
HHUS average time	19 min	15–20 min	20 min

## Discussion

Considering the guiding question of this review, the medical time dedicated to the two methods of breast evaluation by ultrasound, we observed with the data from the studies included in this review that less medical time was spent on the automated method in most studies, but with few studies directly comparing both methods regarding the specific question of medical time dedicated to each one of them. 1 2 10 11 13 17 19 21 23

Of the nine selected studies, seven brought only time information for one of the methods, either because the measurement of this time had not been included in the methodology of these studies or because the comparison between the two methods was not the objective of these researches. 1 10 13 17 19 21 23

The two studies that presented the time for both methods differed in their conclusions regarding medical time. 11 18 Tutar et al. included 340 patients in a cross-sectional study in which the average time for interpretation of automated ultrasound images was 14.5 minutes, greater than the average of 12.5 minutes observed for conventional ultrasound, with data reported descriptively. The authors attributed this result to the fact that they recorded all the lesions observed and analyzed all the images of the coronal, transverse, and longitudinal planes of each of the views (anteroposterior, lateral, and medial) obtained for each of the breasts in the automated ultrasound. 18

However, a similar analysis was cited in the methodology of studies that measured medical time for interpretation of automated images. 10 13 17 21 23 The study by Skaane et al. stands out, with results that reinforce the observation that the number of findings interferes with the time required for image analysis. For the analysis of the images of both breasts, they obtained, on average, 9 minutes, and, considering the time of each breast individually, normal breasts or breasts with cysts required an average of 4 minutes, while breasts with probably benign nodules required 4.8 minutes, and breasts with suspicious findings for cancer required an average of 5.3 minutes. 10

The other study that uses time data for both methods also has a cross-sectional design, including 440 patients. This study brings in its methodology the particularity of the different time of execution of conventional ultrasound by breast radiologists (average time of 7 minutes and 45 seconds) and by radiologists not specialized in breast imaging (average time of 4 minutes and 15 seconds). Automated ultrasound data were interpreted only by breast radiologists, in an average time of 4 minutes and 25 seconds. The difference between the means of the breast radiologists was analyzed for both methods using the t-Student test and was considered statistically significant ( *p* < 0.001). 11

Philadelpho et al. (2021) and Tutar et al. (2020) included patients with dense breasts in breast cancer screening in their studies. High-risk patients and those who had already been diagnosed and were being followed up were excluded, thus sampling a population whose exams tend to present fewer findings. Therefore, Philadelpho et al. (2021) obtained data similar to those of Wilczek et al. (2016) (Easy Study) and Jiang et al. (2018), who also sampled low-risk populations for breast cancer. 11 13 18 21

Skaane et al. (2015) and Vourtsis and Kachulis (2017) did not restrict the participation of patients and, thus, sampled more heterogeneous populations, with the possibility of a greater number of ultrasound findings; however, they obtained very different time means. Skaane et al. (2015) describes an average of 9 minutes among 90 participants, while Vourtsis and Kachulis (2017) describe a much lower average of 3 minutes, but with a much larger number of participants, 1,886. 10 17

For conventional ultrasound, low- and high-risk women were represented, in a non-comparative way with the automated method, in only 3 studies, which described similar time averages, between 15 and 20 minutes. However, Berg et al. (2008) and ACRIN 6666, and Chang et al. (2015) bring into their methodology the axillary evaluation as part of the exam, this time being added to the total time of the conventional exam, similar to the evaluation made by Tutar et al. (2020). 1 18 19 However, Phalak et al. (2018) obtained an average of 20 minutes without axillary evaluation, with the particularity of the examination being performed by technologists and reviewed by radiologists, as authorized in Texas, the state where the study was carried out. 2

Thus, we observed that even considering only the time variable, many factors are associated and interfere with its measurement, probably explaining the variability of data obtained in the literature for both conventional and automated methods of breast ultrasound `evaluation.

As a limitation of this review, we have the small number of studies that evaluated the medical time in both methods, the fact that they are studies with a lower level of evidence, level 4, and the question that only one of them included a statistical analysis of the difference between the averages obtained for the time variable.

These observations suggest that the comparison of the times spent by the physician with each of the methods needs to be better evaluated in experimental studies, with a larger number of patients, which could allow a better evaluation of the potential of automated ultrasound in optimizing medical time.

## Conclusion

In our integrative literature review, the automation of breast ultrasound represents a possible strategy for optimizing the medical time dedicated to the method, but it needs to be better evaluated in comparative studies between both methods, with a methodology aimed at the specific investigation of this potentiality.
